# Lecithin-Bound Iodine Prevents Disruption of Tight Junctions of Retinal Pigment Epithelial Cells under Hypoxic Stress

**DOI:** 10.1155/2016/9292346

**Published:** 2016-05-31

**Authors:** Masahiko Sugimoto, Mineo Kondo

**Affiliations:** Department of Ophthalmology, Mie University, Graduate School of Medicine, 2-174 Edobashi, Tsu, Mie 514-8507, Japan

## Abstract

*Aim*. We investigated whether lecithin-bound iodine (LBI) can protect the integrity of tight junctions of retinal pigment epithelial cells from hypoxia.* Method*. Cultured human retinal pigment epithelial (ARPE-19) cells were pretreated with LBI. To mimic hypoxic conditions, cells were incubated with CoCl_2_. We compared the integrity of the tight junctions (TJs) of control to cells with either LBI alone, CoCl_2_ alone, or LBI + CoCl_2_. The levels of cytokines in the conditioned media were also determined.* Results*. Significant decrease in the zonula occludens-1 (ZO-1) intensity in the CoCl_2_ group compared to the control (5787.7 ± 4126.4 in CoCl_2_ group versus 29244.6 ± 2981.2 in control; average ± standard deviation). But the decrease was not significant in the LBI + CoCl_2_ (27189.0 ± 11231.1). The levels of monocyte chemoattractant protein-1 (MCP-1) and Chemokine (C-C Motif) Ligand 11 (CCL-11) were significantly higher in the CoCl_2_ than in the control (340.8 ± 43.3 versus 279.7 ± 68.3 pg/mL for MCP-1, and 15.2 ± 12.9 versus 12.5 ± 6.1 pg/mL for CCL-11. With LBI pretreatment, the levels of both cytokines were decreased to 182.6 ± 23.8 (MCP-1) and 5.46 ± 1.9 pg/mL for CCL-11). Blockade of MCP-1 or CCL-11 also shows similar result representing TJ protection from hypoxic stress.* Conclusions*. LBI results in a protective action from hypoxia.

## 1. Introduction

Diabetic retinopathy (DR) is a leading cause of visual impairment and blindness in developed countries. The decrease in vision is due to diabetic macular edema (DME) and proliferative diabetic retinopathy (PDR) [1, 2]. The control of the blood glucose level, vitreous surgery, and photocoagulation are the major treatments used to prevent DR from progressing to the PDR stage. Currently, intravitreal injections of antivascular endothelium growth factor (VEGF) or steroids have become the primary therapy for DME [3–5]. However, the use of a single therapeutic agent is not effective in all cases, and additional treatments or different agents are needed.

The blood retinal barrier (BRB) consists of two anatomical parts; the inner BRB is located within the endothelial cells of the retinal capillaries, and the outer BRB is located between the retinal pigment epithelial (RPE) cells [6]. An intact BRB is required for an efficient and regulated control of fluids in the subretinal space and for the maintenance of healthy RPE and retinal cells [7]. A breakdown of the outer BRB results in an increase in the paracellular permeability between the RPE cells, and the leakage can cause retinal edema [8].

The tight junctions (TJs) of the RPE cells are intercellular junctions located at the apical ends of the RPE cells, and they are integral structural components of the BRB. TJs are made up of three TJ related proteins, for example, the zonula occludens-1 (ZO-1) [9], occludin, and claudin. Alterations of the conformation of these proteins lead to a breakdown of the BRB.

Recent studies have shown that interactions between inflammatory cells and retinal cells are critical for the development of intraocular neovascularization [10–12]. The results of these studies also demonstrated that, in eyes with PDR, an elevation of VEGF was significantly correlated with the levels of several cytokines including interleukin- (IL-) 6, IL-8, and the monocyte chemoattractant protein-1 (MCP-1). Another study showed that the levels of these cytokines were also strongly correlated with each other which suggest that there are common pathways involved in the inflammatory processes [13]. In addition, various cytokines have been shown to be related to the maintenance of the conformation of the TJ proteins [14].

Lecithin-bound iodine (LBI, Jolethin®, Daiichi Pharmaceutical Co., Tokyo, Japan) has been used clinically to reduce the antigen-induced immune responses in children with bronchial asthma. LBI acts on the peripheral blood mononuclear cells and downregulates the IL-4-induced IgE synthesis, which suggests that LBI have anti-inflammatory properties [15, 16]. In patients with eye diseases, LBI has been used for the absorption of retinal or vitreous bleeding, vitreous opacities, and improvement of central serous choroidopathy. However, its mechanism of action has not been definitively determined.

The purpose of this study was to determine whether LBI will alter the integrity of the TJs of ARPE-19 cells in culture.

## 2. Materials and Methods

### 2.1. Lecithin-Bound Iodine (LBI)

LBI (Jolethin, Daiichi Pharmaceutical Co., Tokyo, Japan) was dissolved in distilled water and diluted to the appropriate concentration for each experiment. An earlier study showed that LBI solutions contain 48.2–50.3% lecithin-iodine, approximately 10% free lecithin, and 40% phosphatidylinositol [16].

Rabbit polyclonal anti-ZO-1 antibody (sc-10804) was purchased from Santa Cruz Biotechnology (Santa Cruz, CA), and Alexa 594 anti-rabbit IgG was purchased from Invitrogen (Molecular Probs®, Eugene, OR). Rabbit anti-MCP-1 antibody (#2029) was purchased from Cell Signaling Technology (Danvers, MA) and rabbit anti-eotaxin (CCL-11) antibody (ab133604) was purchased from Abcam (Cambridge, MA).

### 2.2. Cell Cultures

Cells from a human retinal pigment epithelium cell line, ARPE-19, were grown in Dulbecco's modified Eagle's medium/Ham's F-12 supplemented with 10% fetal bovine serum (FBS, Hyclone Laboratories, Inc., Logan, UT), 50 units/mL penicillin, and 50 *µ*g/mL streptomycin in an air-5% CO_2_ atmosphere with constant humidity.

### 2.3. Cell Treatment

Prior to the experiments, cells were placed in serum-free media and pretreated with 50 *µ*g/mL of LBI or 250–500 pg/mL of antibody (anti-MCP-1 antibody and anti-Chemokine (C-C Motif) Ligand-11 (CCL-11) antibody) for 24 h. To mimic hypoxic conditions, ARPE-19 cells were incubated for 4 h with 100 *µ*M of CoCl_2_ (Sigma, St. Louis, MO), which is a chemical hypoxia-inducing agent [17]. After the incubation, the media and cover slips were collected for use in the experiments. Optimum concentration of LBI was defined as 50 *µ*g/mL because over 100 *µ*g/mL of LBI seems to be toxic (data not shown).

### 2.4. Immunofluorescence Histochemistry

For the immunofluorescence studies, cells were grown to a density of 2 × 10^4^ cells/mL on 12 mm cover slips and fixed in 10% tricarboxylic acid for 10 min at 4°C. They were then treated with 0.5% triton X-100 for 15 min. To detect the presence of ZO-1, the cultured RPE cells were exposed to rabbit polyclonal anti-ZO-1 antibody as the primary antibody and Alexa 594 anti-rabbit IgG as the secondary antibody.

Samples were examined and photographed with a fluorescence microscope (BZ-9000; Keyence, Osaka, Japan, and Eclipse 50i, Nikon, Tokyo, Japan), and the intensity of fluorescence was quantified with a BZ-II Analyzer (Keyence).

### 2.5. Enzyme-Linked Immunosorbent Assay (ELISA)

The levels of the bioactive molecules in the conditioned medium were determined by human IL-6, IL-8, and MCP-1 ELISA kits (R&D Systems, Minneapolis, MN) and CCL-11 ELISA kit (Biosensis, Thebarton, South Australia, Australia). Each assay was performed in accordance with the manufacturer's instructions.

### 2.6. Statistical Analyses

All experiments were repeated at least three times and values are presented as the means ± standard deviations. Data were analyzed by two-way nonrepeated analysis of variance (ANOVA) followed by Bonferroni post hoc tests for the comparison of the means. Statistical significance was set at *P* < 0.05.

## 3. Results

### 3.1. Lecithin-Bound Iodine Protects Conformation of Tight Junctions Proteins from Hypoxic Stress

**Figure 1:**
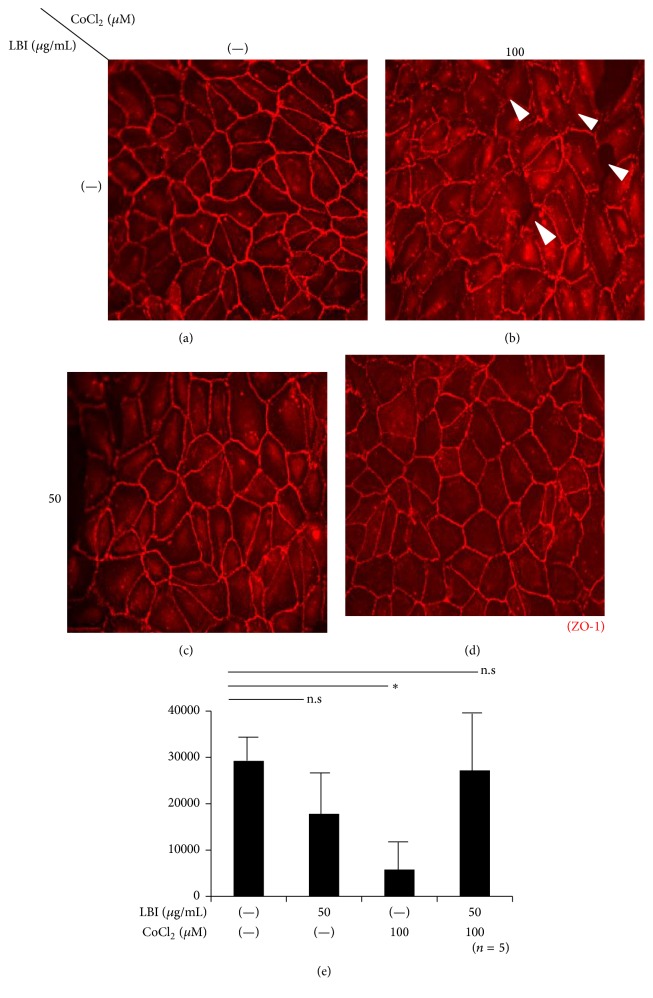
Photomicrographs of ARPE-19 cells in culture immunostained for ZO-1, a TJ protein. The findings demonstrate that LBI protects TJs from disruption by hypoxia-induced stress. Immunofluorescence microscopic images showing the presence of ZO-1 (red) surrounding the ARPE-19 cells in culture. Exposure of the cells to CoCl_2_ causes a disruption of the TJs ((a), (b) arrow head). When the CoCl_2_-exposed ARPE-19 cells were pretreated with LBI, there were no significant differences in the ZO-1 pattern from that of the control ((c), (d)). The integrity of the TJs was also estimated using signal intensity measurements (*n* = 5) (e). Although there was a significant decrease in the signal intensity after the addition of CoCl_2_ compared to that of the controls, the difference was not significant when the cells were pretreated with LBI prior to the addition of CoCl_2_. ^*∗*^ *P* < 0.05, nonrepeated ANOVA. LBI, lecithin-bound iodine; n.s, not significant; TJs, tight junctions; ZO-1, zonular occludin-1.

We first determined whether the LBI pretreatment affected the changes in the conformation of the TJ proteins caused by the hypoxic stress induced by CoCl_2_. Immunofluorescence microscopy showed that there appeared to be a disruption of TJs with CoCl_2_ treatment (Figures 1(a) and 1(b)). When the ARPE-19 cells were pretreated with LBI before the addition of CoCl_2_, the disruption of the tight junctions was not detected (Figures 1(c) and 1(d)). The signal intensity measurements showed that there was a significant decrease with the CoCl_2_ addition compared to the control. But there was no significant change in the LBI pretreated group (29244.6 ± 2981.2 in controls; 5787.7 ± 4126.4 with CoCl_2_; and 27189.0 ± 11231.1 with CoCl_2_ after LBI pretreatment; *P* < 0.05, nonrepeated ANOVA, *n* = 5, Figure 1(e)). Because disruption of TJs is detected as a decrease of signal intensity, these results indicate that LBI pretreatment can protect the TJs of the outer BRB from hypoxic stress.

### 3.2. Hypoxia Induces Increases in MCP-1 and CCL-11 Which Is Suppressed by LBI Pretreatment

**Figure 2:**
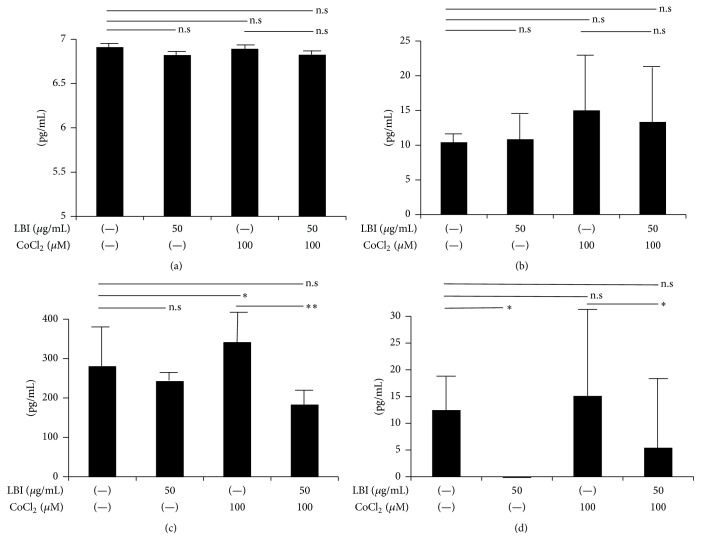
Hypoxia induces increases in MCP-1 and CCL-11 which is depressed by LBI pretreatment. ELISA was used to measure several cytokines in the conditioned culture media (*n* = 5). No significant changes are observed for IL-8 with the addition of CoCl_2_ or LBI pretreatment (a). An increase in the IL-6 was observed after the addition of CoCl_2_ while IL-6 decreases with the addition of CoCl_2_ after LBI pretreatment (b). The significant increase in the MCP-1 observed after the addition of CoCl_2_. But it disappeared with LBI pretreatment (c). A similar tendency was also observed for CCL-11 (d). ^*∗*^ *P* < 0.05, ^*∗∗*^ *P* < 0.01, nonrepeated ANOVA. CCL-11, Chemokine (C-C Motif) Ligand 11; IL, interleukin; LBI, lecithin-bound iodine; MCP-1, monocyte chemoattractant protein-1. n.s, not significant.

Because LBI pretreatment protected the conformation of the TJ proteins from hypoxic stress, we hypothesized that LBI will block different inflammatory molecules that are secreted from cells during hypoxia. The results of the ELISA measurements of the conditioned culture media (*n* = 5) indicated that IL-8 was not significantly changed after the addition of CoCl_2_ or after pretreatment with LBI (control, 6.9 ± 0.1; with CoCl_2_, 6.8 ± 0.1; and with CoCl_2_ after LBI pretreatment, 6.9 ± 0.1 pg/mL; Figure 2(a)). Although the level of IL-6 was increased after addition of CoCl_2_ which was decreased by LBI pretreatment, these changes were not significant (control, 10.4 ± 1.6; CoCl_2_ addition, 15.0 ± 6.7; and CoCl_2_ addition after LBI pretreatment, 13.3 ± 7.6 pg/mL; Figure 2(b)).

The level of MCP-1 increased significantly after the addition of CoCl_2_, and it was significantly decreased after the addition of CoCl_2_ after LBI pretreatment (control, 279.7 ± 68.3; with CoCl_2_, 340.8 ± 43.3; and with CoCl_2_ after LBI pretreatment, 182.6 ± 23.8 pg/mL, *P* < 0.05; nonrepeated ANOVA; Figure 2(c)). A similar tendency was observed for CCL-11 (control, 12.5 ± 6.1; with CoCl_2_, 15.2 ± 12.9; and with CoCl_2_ after LBI pretreatment, 5.46 ± 1.9 pg/mL; *P* < 0.05; nonrepeated ANOVA; Figure 2(d)). Though significant increase was not seen, it tended to increase after CoCl_2_. This endogenous secretion of CCL-11 was suppressed only after LBI pretreatment. These results indicated that there is an increase of the MCP-1 and CCL-11 secretion under hypoxic stress, and all are suppressed by LBI pretreatment.

### 3.3. Blocking of MCP-1 and CCL-11 Protects Conformation of Tight Junctions Proteins from Hypoxic Stress

**Figure 3:**
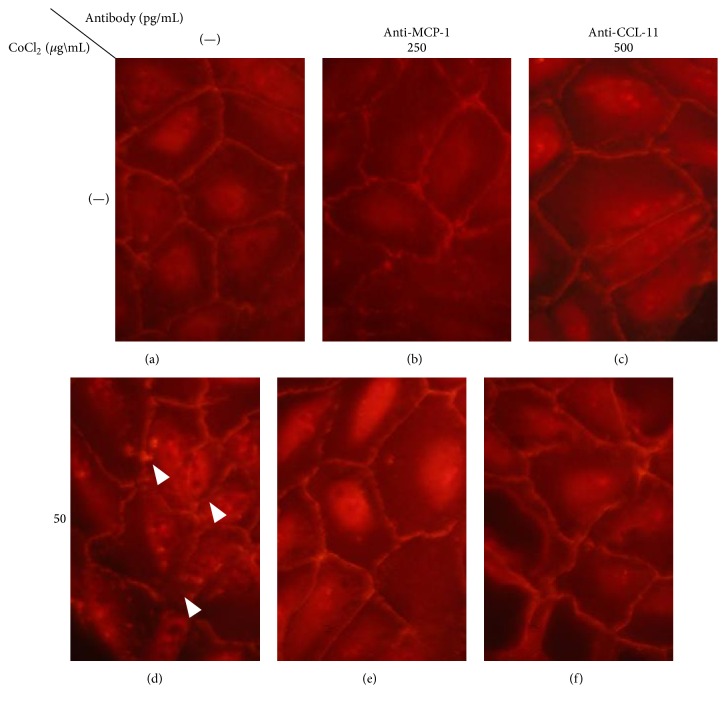
Hypoxia-induced tight junction disruption is also suppressed by blockade of MCP-1 or CCL-11. Exposure on ARPE-19 cells to CoCl_2_ causes TJ disruption ((d) arrow head). When the cells were pretreated with anti-MCP-1 or CCL-11 antibody, these changes were reduced in the ZO-1 pattern compared to that of the control ((e), (f)). Thus, blockade of MCP-1 and CCL-11 can protect conformation of TJs from hypoxic stress. CCL-11, Chemokine (C-C Motif) Ligand 11; IL, interleukin; LBI, lecithin-bound iodine; MCP-1, monocyte chemoattractant protein-1; TJs, tight junctions; ZO-1, zonular occludin-1.

 To confirm that LBI effectiveness is related to suppression of MCP-1 or CCL-11, cells were pretreated with antibodies to block each cytokine. Before CoCl_2_ addition, cells were pretreated with anti-MCP-1 or anti-CCL-11 antibody for 24 hrs. The TJ disruption from hypoxic stress was reduced with pretreatment (Figures 3(e) and 3(f)). These results indicated that both MCP-1 and CCL-11 were important to protect TJs from hypoxic stress and support the effectiveness of LBI.

### 3.4. LBI Pretreatment Protects Conformation of Tight Junctions Proteins from VEGF

**Figure 4:**
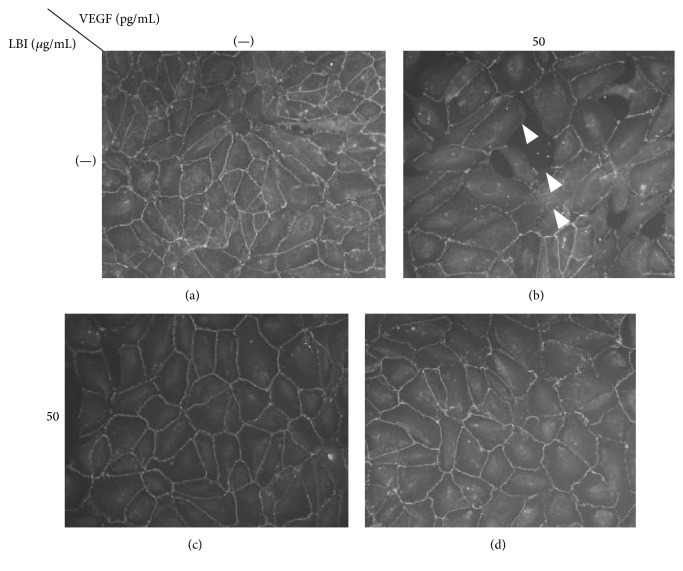
LBI can protect tight junctions from VEGF. Fifty pg/mL of VEGF was added to culture medium for 6 hrs. ARPE-19 cells ((a)–(d)) were stained with anti-ZO-1 antibody and TJs conformation was evaluated. Note that TJs breakdown was observed after VEGF ((b) arrows). LBI pretreatment before VEGF addition could protect TJs from disruption (d). LBI, lecithin-bound iodine; TJs, tight junctions; VEGF; vascular endothelium growth factor; ZO-1, zonular occludin-1.

VEGF is a major antigen factor and is biogenic permeability factor which can increase the vascular permeability endothelial cell-cell junctions [18]. Finally, to confirm that LBI pretreatment can protect TJs from direct VEGF induced stress, after LBI pretreatment, 50 pg/mL of VEGF was added to induce TJ damage. After 6 hours, VEGF induced TJ disruption occurred (Figure 4(b)). But after pretreatment of LBI, this disruption was decreased which indicates that LBI can also protect TJs from VEGF induced damage.

## 4. Discussion

Anti-VEGF therapy has become the standard treatment for macular edema including DME. But there still remain patients who do not respond to the anti-VEGF therapy [3] and who had a reduction of their vision even after therapy [5]. It must also be noted that the cost of anti-VEGF therapy is quite high and continuous injections to control the DME can become an economic issue [19].

Combination therapies have also been proposed that can enhance the efficacy [20] and may minimize the cost of anti-VEGF therapies. For example, photocoagulation can extend the interval of anti-VEGF injections resulting in a reduction in the number of injections [21, 22]. If new drugs or treatments can be approved, this will make it easier and less expensive to use combination therapies. Moreover, adjunctive therapies that include oral kallidinogenase could also be used for combined therapy [23, 24].

Our results showed that the hypoxic stress induced by exposure of ARPE-19 cells to CoCl_2_ caused a disruption of the tight junctions and that LBI pretreatment can protect the TJs from the hypoxic stress. Hypoxic stress also enhanced the secretion of MCP-1 and CCL-11, and this enhancement can be suppressed by LBI pretreatment.

Hypoxia has been reported to produce alterations in the tight junction proteins that are correlated with the vascular permeability increases [25]. The integrity of the BRB is dependent on TJ-associated proteins such as occludin, claudin, and ZO-1 [26]. In particular, ZO-1 plays an important role in helping to maintain its integrity [27]. The function of ZO-1 is to link the transmembrane protein, occludin, to the actin cytoskeleton [28]. Under hypoxic conditions, the phosphorylation of ZO-1 is enhanced which is related to the damage of the tight junctions [29].

Various cytokines including chemokines are also involved in maintaining the conformation of the TJs [14]. MCP-1 is associated with a breakdown of the BRB [30–32], and it also induces a disruption of the TJs by the caveolae that are dedicated to the internalization of the TJ proteins, such as ZO-1 [33]. Because MCP-1 is also increased in the vitreous of patients with DR, it probably plays an important role in the progression of DR and DME [13]. In the photocoagulation-induced mouse model of retinal neovascularization, it was reported that an increase in MCP-1 contributes to the postischemic inflammation and DR progression [34, 35]. MCP-1 has been recently postulated to have a direct effect on angiogenesis even though there is no link with macrophage recruitment [36]. CCL-11 also promotes the recruitment of vascular endothelial cells and relates the conformation of the tight junctions [37]. Interestingly, there is also an increase of CCL-11 in the proliferative membranes obtained from eyes with PDR [38]. So these cytokines relate to ZO-1 and maintain tight junctions on diabetic retina.

In the photocoagulation-induced retinal neovascularization or light-exposed mouse models, the levels of not only MCP-1 but also CCL-11 are increased in the RPE [39–41]. These increases were related to the remodeling of F-actin that occurred after light damage and caused proinflammatory changes in the RPE cells. These findings support our results that showed that the hypoxia-induced stress affected the expression of cytokines especially MCP-1 and CCL-11. The suppression of these cytokines by LBI leads to the protection of these cells from disruption of the tight junctions. Steroids are also commonly used for DME therapy because their anti-inflammatory property helps to protect the tissues from neovascularization or vascular permeability increases. Similarly, the effect of LBI on the suppression of the postischemic inflammation may be an additional contributor when used as either a primary or adjunctive DME therapy.

Mainly in Japan and China, LBI has been approved for the treatment of various retinal diseases including central serous chorioretinopathy, vitreous hemorrhage, or vitreous opacity for a long time [42]. In addition, both lecithin and iodine are commonly used in food materials indicating the safety of LBI. Thus, the possibility exists that LBI can be even used as a safe adjunctive therapy with an anti-VEGF drug to treat DME. Further investigations into the use of LBI in conjunction with anti-VEGF therapy need to be undertaken to determine the effectiveness of LBI in preventing the development or progression of DME.

Our findings showed that LBI pretreatment reduced the levels of MCP-1 and CCL-11 and blockage of them also results in protection of TJs. However, no significant changes were detected in the levels of IL-6 and IL-8, which are the major cytokines related to neovascularization. In ischemic retinas, IL-8 causes increases in the VEGF and MCP-1 levels but it is dependent on TNF-*α* [43]. Because IL-8 has been shown to be secreted from either monocytes or macrophages [44] and because our experiments were limited to ARPE-19 cells in culture, there is the possibility that the results may not hold under* in situ* conditions. Though we observed that vascular permeability improved after oral LBI administration for diabetic mice model (data not shown), it is not clear whether this is the result of hypoxia improvement the same as we show here using ARPE-19. In addition, LBI pretreatment can also protect TJs from direct VEGF induced damage which results in TJ disruption. It also indicates that LBI may be useful for DME treatment. Further investigations will need to be undertaken to definitively explain these phenomena.

In conclusion, our data demonstrate the therapeutic potential of LBI for protecting the integrity of the tight junctions from hypoxia-induced stress. These data suggest a new aspect on the clinical use of LBI in the treatment of DME.
